# Elevated circulating growth differentiation factor 15 is related to decreased heart rate variability in chronic kidney disease patients

**DOI:** 10.1080/0886022X.2021.1880938

**Published:** 2021-02-10

**Authors:** Lulu Wang, Jing Luo, Wenjin Liu, Xiaoqin Huang, Jie Xu, Yang Zhou, Lei Jiang, Junwei Yang

**Affiliations:** a Center for Kidney Disease, 2nd Affiliated Hospital, Nanjing Medical University, Nanjing, China; b Department of Radiology, University of Washington, Seattle, WA, USA

**Keywords:** GDF15, heart rate variability, chronic kidney disease

## Abstract

**Background:**

Growth differentiation factor 15(GDF15) is a distant member of the superfamily of the transforming growth factor beta (TGF-β). It has been established that increased GDF15 levels are associated with an increased risk of cardiovascular disease. However, the detail effect of GDF15 on cardiovascular system in patients with chronic kidney disease (CKD) needs detail analysis.

**Methods:**

Patients with CKD who did not need dialysis were enrolled in the study. Blood pressure (BP), endothelial function, pulse wave velocity (PWV) and heart rate variability (HRV) were taken in all subjects. Plasma GDF15 concentration was measured by an enzyme-linked immunosorbent assay.

**Results:**

Among the 355 participants, the mean age was 57.4 (±14.2) years old and the mean estimated glomerular filtration rate (eGFR) was 50.1 (±33.2) mL/min/1.73m^2^. The average plasma GDF15 level was 1394.7 (±610.1) pg/mL. Higher GDF15 concentrations were significantly associated with decreased eGFR and increased urine protein-to-creatinine ratio (uPCR). In multivariable models, after adjusting for potential confounders, plasma GDF15 has negative concerning with HRV parameters including the standard deviation of the normal-to-normal (NN) interval (SDNN), the square root of the mean of the sum of the squares of differences between adjacent NN intervals (RMSSD) and Triangular Index.

**Conclusion:**

We observed there was a link between increased plasma of GDF15 and decreased HRV. The mechanisms and prediction of GDF15 in the cardiovascular disease with CKD needs further discussion and study.

## Introduction

It is well known that patients with chronic kidney disease (CKD) are at a high risk of cardiovascular disease and death [1]. A majority of patients might even die of cardiovascular disease (CVD) rather than progress to end-stage renal disease (ESRD) [2]. The pathogenesis of cardiovascular disease in CKD is complex. Substantial CVD risk exists in CKD patients despite the fact that we have controlled traditional risk factors, such as diabetes and hypertension. Identification of the different pathophysiological pathways involved in CVD is a significant step forward in developing potentially effective therapies.

Growth differentiation factor 15 (GDF15) is a protein of the transforming growth factor beta (TGFβ) superfamily. It was first separated from a U937 subtraction cDNA library and originally named macrophage inhibitory factor 1 (MIC-1) [3]. It was subsequently given the official designation of growth differentiation factor 15 (GDF15) [4] and is also known as prostate differentiation factor, placental TGF-beta, and nonsteroidal anti-inflammatory drug-activated protein-1 [5]. Human GDF15 is localized on chromosome 19 and consists of two exons separated by an intron. GDF15 is first synthesized as an inactive precursor protein with 308 amino acids with a 29-amino-acid signal peptide at the N-terminal region. It is then separated by furin at an RXXR site to yield a propeptide and a mature peptide; the latter is secreted as a dimeric protein of 224 amino acids with a molecular weight of approximately 25 KDa [6,7] and is regarded as the active form of GDF15. GDF15 can specifically bind to GDNF family receptor α-like (GFRAL) with high affinity and forms a complex with RET, the transmembrane tyrosine kinase coreceptor, which subsequently activates intracellular signaling pathways, thereby facilitating several biological effects [8,9]. Previous studies have suggested a link between GDF15 and CVD. A study by Wollert found higher plasma GDF15 levels in patients with cardiovascular events, and the effect was an independent cardiovascular risk factor [10]. However, animal experiments led to diverse conclusions that the protein could inhibit the development of atherosclerosis [11].

Currently, many reports have focused on the effects of GDF15 on cardiac function. There is a paucity of data regarding the association between GDF15 levels and cardiovascular alterations in patients with CKD, which we sought to explore in this study. We hypothesized that plasma GDF15 levels would be higher in patients with CKD, which could have probable effects on arterial stiffness, endothelial function, and heart rate variability (HRV).

## Materials and methods

### Study population

A total of 355 patients were recruited for this study. We included all adult patients (over 18 years old), who had a CKD diagnosis according to the National Kidney Foundation Kidney Disease Outcomes Quality Initiative Guidelines [12]. Exclusion criteria of the study were renal replacement therapy or malignant hypertension (systolic blood pressure ≥ 180 mm Hg or diastolic blood pressure (DBP) ≥ 110 mm Hg), malignancy or any other unstable situation making them unsuitable for examination. The study was approved by the institutional research ethics committee of The Second Affiliated Hospital of Nanjing Medical University (ID: [2015]KY052), and all participants were able to give informed consent.

### Pulse wave velocity (PWV) and peripheral arterial tonometry (PAT)

The pulse wave velocity (PWV) and peripheral arterial tonometry (PAT) measurements were obtained as described previously [13]. Carotid-femoral PWV (cfPWV) and carotid–radial pulse wave velocity (crPWV) were detected using the Complior Analyzer device (Artech Medical, France). Reactive hyperemia index (RHI), which reflects endothelial function, and HRV, which assesses autonomic nervous function, were measured with the Endo-PAT 2000 (Itamar Medical Inc, Israel) by peripheral arterial tonometry after PWV measurement. The RHI is the ratio of amplitude of the signal after cuff deflation divided by that before cuff inflation, indexed to the contralateral arm. The time-domain measures of HRV include the standard deviation of the normal-to-normal (NN) interval (SDNN), the square root of the mean of the sum of the squares of differences between adjacent NN intervals (RMSSD) and Triangular Index, which is the total number of all NN intervals divided by the height of the histogram of all NN intervals measured on a discrete scale with bins of 7.8125 ms. The frequency domain index low to high frequency ratio (LF/HF) measurement is the ratio of low frequency to high frequency components.

### Clinical information

General demographic and medical information were obtained through an inquiry of history and review of medical records. We defined previous history of CVD as any of following: acute myocardial infarction, ischemic or hemorrhagic stroke, atrial fibrillation, coronary heart disease other than myocardial infarction, chronic heart failure and other types confirmed by the research staff. A sample of fasting blood or first voided morning urine was sent to the local laboratory department for examination. Blood routine biochemical tests included hemoglobin, C-reactive protein (CRP), total cholesterol, triglyceride, high-density lipoprotein cholesterol (HDL-C), low-density lipoprotein cholesterol (LDL-C), calcium, and phosphorus. The protein-to-creatinine ratio (PCR) in urine was examined. Estimated glomerular filtration rate (eGFR) was calculated using the CKD-EPI formula.

### Plasma GDF15

Fasting blood samples were collected into ethylenediaminetetraacetic acid (EDTA)-treated tubes and frozen at −80 °C until measurement. Plasma GDF15 was measured by an enzyme-linked immunosorbent assay (ELISA) (DY957, R&D Systems) according to manufacturer’s protocol. The assay range is 7.81–500 pg/mL and all plasma samples were measured at a dilution of 1:10.

### Statistical analyses

Continuous variables were expressed as the mean ± standard deviation or median (interquartile range) and categorical variables were expressed as counts (%). Numerical variables with a skewed distribution were logarithmically transformed for further analysis if needed. The relationship between GDF15 and renal function or proteinuria was determined using Pearson’s correlation analysis. A multiple linear regression model was constructed to confirm the relationship between GDF15 and cardiovascular risk factors. Variables such as uPCR, SDNN, RMSSD, Triangular Index and LF/HF were logarithmically transformed in regression analysis due to their skewed distribution. All statistical analyses were performed using SPSS 19.0 (IBM SPSS, Chicago, IL), and *p* values less than .05 were considered statistically significant.

## Results

A total of 355 participants were included in the study. Table 1 displays an overview of the demographic and general medical information of the patients. Briefly, the mean eGFR was 50.1 (±33.2) mL/min/1.73 m^2^ and median urine protein-to-creatinine ratio (uPCR) was 150 mg/g.

**Table 1.** Characteristics of the Study Population (*N* = 355).

	Mean ± SD / Number (%)
Age, years	57.4 ± 14.2
BMI, kg/m^2^	25.8 ± 4.0
Male	224 (63.1%)
Current Smoker	93 (26.2%)
Diabetes	158 (44.5%)
Cardiovascular disease	82 (23.1%)
Etiology	
Diabetic kidney disease	113 (31.8%)
Hypertensive nephropathy	37 (10.4%)
Glomerulonephritis	121 (34.1%)
Others	37 (10.4%)
Undetermined	47 (13.2%)
Statins	152 (42.8%)
Epo/ iron supplements	91(25.6%)
Systolic Blood pressure, mm Hg	137.0 ± 20.4
Diastolic Blood pressure, mm Hg	84.5 ± 11.5
Heart rate, bpm	74.5 ± 12.7
Hemoglobin, g/L	117.5 ± 24.4
CRP, mg/L	0.9(0.5—3.2)
Total cholesterol, mmol/L	4.7 ± 2.1
Triglycerides, mmol/L	2.1 ± 1.7
HDL cholesterol, mmol/L	1.1 ± 0.5
LDL cholesterol, mmol/L	3.0 ± 1.6
Calcium, mmol/L	2.1 ± 0.2
Phosphorus, mmol/L	1.2 ± 0.3
Urine PCR, mg/g	150.0 (40.5—358.5)
eGFR, mL/min/1.73m^2^	50.1 ± 33.2
cfPWV, m/s	10.4 ± 3.4
RHI	2.0 ± 0.6

BMI: body mass index; EPO: erythropoietin; CRP: C-reactive protein; HDL-C: high-density lipoprotein cholesterol; LDL-C: low-density lipoprotein cholesterol; PCR: protein to creatinine ratio; eGFR: estimated glomerular filtration rate; cfPWV: carotid–radial pulse wave velocity; RHI: Reactive hyperemia index.

The average plasma GDF15 level was 1394.7 (±610.1) pg/mL. We first evaluate GDF15 concentration within several subgroups, including diabetes mellitus (no vs. yes), previous history of cardiovascular disease (no vs. yes), different CKD stages (CKD stage 1 + 2 group vs. 3 vs. 4 + 5) and uPCRs (uPCR <30mg/g vs. 30–300mg/g vs. >300mg/g). As shown in Table 2, there was a marked difference of plasma GDF15 level across these subgroups (all *p <* .05).

**Table 2.** GDF15 level in all patients and subgroups.

	Mean ± SD, pg/mL	*p*
All (*n* = 339)	1394.7 ± 610.1	
Diabetes mellitus		<.001
No (*n* = 190)	1254.65 ± 616.24	
Yes (*n* = 149)	1573.28 ± 554.84	
Previous history of CVD		.03
No (*n* = 262)	1360.04 ± 634.05	
Yes (*n* = 77)	1512.64 ± 506.51	
CKD stages		<.001
CKD stage 1 + 2 group (*n* = 119)	1019.3 ± 532.7	
CKD stage 3 group (*n* = 106)	1250.8 ± 414.6	
CKD stage 4 + 5 group (*n* = 114)	1920.4 ± 458.9	
uPCR stages		<.001
<30mg/g (*n* = 65)	1051.5 ± 449.0	
30mg/g–300mg/g (*n* = 172)	1329.2 ± 609.3	
>300mg/g (*n* = 97)	1725.1 ± 535.4	

**Figure 1.**
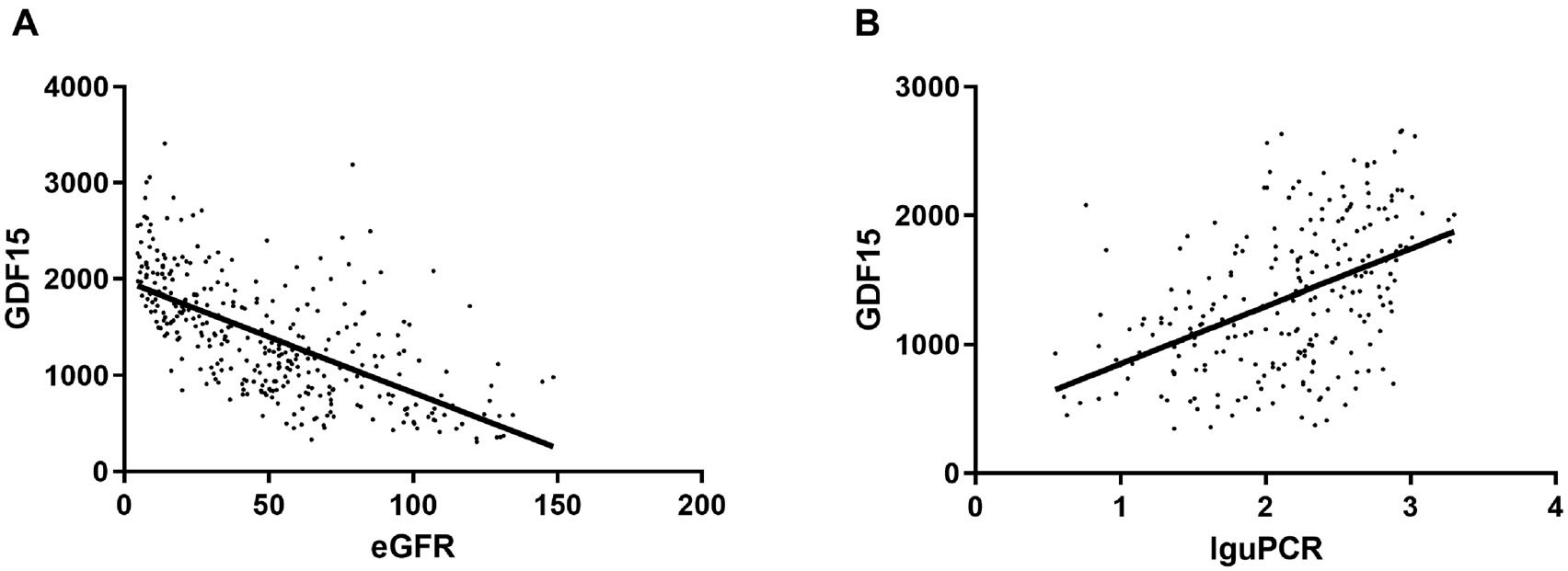
Correlation between GDF15 and eGFR and urine protein-to-creatinine ratio (*n* = 355) (A) GDF15 is significantly correlated with eGFR. (B) GDF15 is negatively significantly correlated with uPCR. Data are displayed as scatterplots. Abbreviations: eGFR estimated glomerular filtration rate, PCR protein-to-creatinine ratio. uPCR was logarithm transformed in correlation analysis.

As shown in Figure 1, there was a significant inverse correlation between GDF15 with eGFR (r = −0.634, *p* < .001) and a significant positive correlation for uPCR (*r* = 0.47, *p* < .001).

Further analysis revealed the correlation between GDF15 and cardiovascular evaluation results in patients with CKD (Table 3). Based on Pearson coefficient correlation analysis, GDF15 was found to be highly related to systolic blood pressure (*r* = 0.303, *p <* .001) and cfPWV (*r* = 0.023, *p* < .001). GDF15 was negatively correlated with SDNN (r = −0.216, *p <* .001), LF/HF (r = −0.173, *p* = .002), and Triangular Index (r = −0.291, *p* < .001).

**Table 3.** Association between GDF15 and cardiovascular evaluation results.

	r	*p*
Systolic blood pressure	0.303	<.001
Diastolic blood pressure	–0.062	.253
cfPWV*	0.023	<.001
LnRHI	0.074	.183
SDNN*	–0.216	<.001
RMSSD*	–0.116	.036
Triangular Index*	–0.291	<.001
LF/HF*	–0.173	.002

cfPWV: carotid–radial pulse wave velocity; RHI: Reactive hyperemia index; SDNN: standard deviation of the normal-to-normal (NN) interval; RMSSD: square root of the mean of the sum of the squares of differences between adjacent NN intervals; LF/HF: low frequency and high frequency ratio.

*These parameters were logarithm transformed in regression analysis due to their skewed distribution. Because of the closely association between PWV and SBP, the association of GDF15 with PWV was analyzed after adjustment for blood pressure.

Finally, we sought to confirm the associations between GDF15 and cardiovascular function evaluation indices using multiple linear regression analysis after adjusting for age, sex, BMI, smoking status, diabetes mellitus, previous history of CVD, antihypertensive drugs, eGFR, CRP, low density lipoprotein, and systolic blood pressure. There was no significant difference between plasma GDF15 and vascular function including cfPWV and LnRHI (all *p* > .05). Plasma GDF15 levels were significantly and negatively associated with SDNN (ß = −0.044, *p* = .026), RMSSD (ß = −0.05, *p* = .029), Triangular Index (ß = −0.038, *p* = .008), and LF/HF (ß = 0.006, *p* = .801). All ß values were calculated for each standard deviation increment of GDF15 (Table 4).

**Table 4.** Associations between cardiovascular evaluation results and GDF15.

	Plasma GDF15					
	Model-1			Model-2		
	ß	95% confidence interval	*p*	ß	95% confidence interval	*p*
CfPWV*	0.024	0.012 − 0.035	<.001	0.007	–0.007 to 0.021	.3
SDNN*	–0.058	–0.088 to −0.028	<.001	–0.044	–0.083 to −0.055	.026
RMSSD*	–0.053	–0.087 to −0.018	.003	–0.05	–0.095 to −0.005	.029
Triangular Index*	–0.053	–0.075 to −0.032	<.001	–0.038	–0.066 to −0.01	.008
LF/HF*	–0.032	–0.07 − 0.006	.099	0.006	–0.044 to 0.056	.801

cfPWV, carotid–radial pulse wave velocity; SDNN, standard deviation of the normal-to-normal (NN) interval; RMSSD, square root of the mean of the sum of the squares of differences between adjacent NN intervals; LF/HF, the ratio of low frequency to high frequency components. Model-1: adjust for age, sex, body mass index. Model-2: adjust for age, sex, body mass index, smoker, diabetes mellitus, previous history of cardiovascular disease, eGFR, CRP, LDL, SBP.

* These parameters were logarithm transformed in regression analysis due to their skewed distribution.

## Discussion

The present study described the association between plasma GDF15 concentration and the cardiovascular system in patients with CKD. We found that GDF15 levels were significantly higher in patients with severe renal impairment manifesting as a low eGFR and high uPCR. In addition, GDF15 was positively associated with systolic blood pressure and cfPWV. Further, multiple regression analysis revealed that increased plasma GDF15 was associated with reduced HRV.

*GDF15* mRNA is highly expressed in the placenta and prostate but expressed in a minimal amount in the kidney under physiological conditions [14]. The expression of *GDF15* mRNA is induced in tissue injury, anoxia, and inflammation with a broad normal range of approximately 200–1200 pg/mL [6,15]. GDF15 is also released from macrophages, vascular smooth muscle cells, endothelial cells, adipocytes, and cardiomyocytes, thereby functioning as an endocrine factor [16,17]. In this study, the average pre-dialysis plasma GDF15 concentration in patients was 1394.7 (±610.1) pg/mL. We also observed that increased levels of GDF15 were related to decreased renal function. Recent clinical studies have established that elevated GDF15 is associated with a two-fold higher risk of deterioration in renal function in two independent CKD cohorts [18]. Consistent with these observations, studies in patients with chronic kidney disease have shown that elevated GDF15 is associated with faster deterioration of kidney function [19]. There are two possible explanations for an increase in the level of GDF15 during renal diseases, either less clearance or increased synthesis of GDF15 or both. Notably, a previous study reported that mature GDF15 is not stored but rapidly secreted in cultured human kidney cells [3]. Moreover, in the context of diabetic renal injury, increased urinary GDF15 is associated with proximal tubule damage [20]. The molecular weight of the protein in circulation was determined to be 25 kDa, which is lower than that of albumin, and it is likely that it is filtered in the glomerulus. These results indicate an association between GDF15 and renal injury and elevated GDF15 may, therefore, worsen the patients’ prognoses.

The production of GDF15 could be more potent under the stressful conditions of impaired kidney function. The mRNA expression of GDF15 was negatively associated with eGFR in the patients of CKD [18]. Other studies have also shown that metabolic acidosis could increase the expression of GDF15 in the outer medullary collecting duct cells [21]. Furthermore, following a kidney transplant, there is a significant decrease in GDF15 at month 12 relative to the baseline levels (1631 pg/mL vs 4744 pg/mL, *p* < .0001) [22]. Therefore, we assumed that the kidney might either be a direct source of GDF15 or increase GDF15 concentrations indirectly through cardiac injury.

We also analyzed the correlation between GDF15 and arterial stiffness and endothelial function in the current study. In the age-, sex-, and BMI-adjusted analysis, higher GDF15 was associated with increased arterial stiffness. The association disappeared after adjusting for potential confounders. We also measured RHI and found that it was not related to GDF15. This seems to differ from the findings in the general population. In a study of participants from the Framingham Offspring Study Cohort (*N* = 1823, mean age 61 years), circulating GDF15 was positively associated with CfPWV and inversely associated with FMD after multivariable adjustment [23]. However, renal function was not determined. This difference may also be a result of methodological differences because of the measurement of RHI and FMD.

Another intriguing finding of this study is the significant association between increased plasma GDF15 levels and reduced HRV. HRV is the beat-to-beat variation in heart rate intervals, which has been proven to be used to assess autonomic imbalances, diseases, and mortality[24]. In the study by Kubota et al., lifetime risks of CVD were significantly increased in the lowest tertile as compared to that in the highest tertile for LF, SDNN, and LF/HF with a median of 24 years of follow-up [25]. This was consistent with a previous report indicating that reduced HRV is linked to an increased risk of MACEs (major adverse cardiovascular events) and hospitalization in dialysis patients [26]. In the early course of CKD, patients have reduced HRV [27]. This is related to impaired reflex control of autonomic activity, cardiovascular remodeling, activation of the renin-angiotensin-aldosterone system, and renal afferents in the context of CKD [28]. Prior literature has shown the significance of circulating GDF15 in cardiovascular risk. In a study by Tuegel et al., each SD increase in GDF-15 levels was related to an estimated 87% higher risk of mortality and 56% higher risk of heart failure in patients with CKD [29]. Breit et al. also found that GDF15 ≥ 7500 pg/mL predicted mortality in ESRD [30]. The mechanistic basis of GDF15 and CVD is not yet known; our study findings could provide promising insights into the exploration of this mechanism. A recent study reported that GFRAL, which is mainly located in the central nervous system, was the only receptor for the mature form of GDF15 [8]. Our findings might reflect that GDF15 expression is compensatory for autonomic dysfunction in patients.

In the past, several investigators have addressed the protective effect of GDF15 on the heart. GDF15 could ameliorate myocardial infarction by inhibiting polymorphonuclear leukocyte infiltration [31]. Moreover, mice overexpressing GDF15 have smaller atherosclerotic lesions when measured at a late stage of the disease [32]. In GDF15-knock-out mice, the expression of adhesion molecules increases along with the atherosclerotic plaque destabilization [16]. Numerous clinical studies have established that high GDF15 levels predict a poor clinical outcome, whereas studies in rodents have shown that GDF15 plays a role in a novel defense mechanism that protects cardiac function. A possible explanation could be that elevated GDF15 levels might be an epiphenomenon and have no role in the pathogenesis of disease, and it could be a result of severe disease rather than the cause. In addition, GDF15 might limit the course of the disease at an early stage [6], but at an advanced stage of the disease, GDF15 receptor antibody could be produced. Moreover, Wang et al. found that hNAG-1 mice show a significant increase in both mean and median life spans in two transgenic lines compared to that in their wild-type counterparts [33]. This suggested that GDF15 may be a regulator of mammalian longevity.

This analysis has several limitations. First, it is a cross-sectional analysis; thus, we cannot establish causality. Second, we did not measure the specific biomarker levels in the tissue, thereby limiting our ability to explore the production of GDF15 in the kidney. Finally, our research was limited to the Chinese population with CKD, and it is thus difficult to extrapolate the results to other ethnic groups.

In summary, our data indicated that in CKD patients, plasma GDF15, which is closely related to impaired renal function and decreased HRV, is elevated. Future studies should explore the mechanistic basis of these alternations and determine if circulating GDF15 could predict cardiovascular outcomes in CKD patients.

## Ethics

All subjects had given their written informed consent and that the study protocol was approved by theinstitutional research ethics committee of The Second Affiliated Hospital of Nanjing Medical University (ID: [2015]KY052).
